# Mechanical Properties of Hybrid Fiber Reinforced Rubber Concrete

**DOI:** 10.3390/ma14206028

**Published:** 2021-10-13

**Authors:** Qiang Su, Jin-Ming Xu, Yong-Dong Wang

**Affiliations:** 1 Department of Civil Engineering, Shanghai University, Shanghai 200444, China; anlida_su@163.com; 2 Shanghai Shancheng Survey & Design Co., Ltd., Shanghai 201803, China; wyondong@163.com

**Keywords:** concrete, mechanical properties, basalt fiber, PVA fiber, rubber, SEM microscopic, EDS quantitative analysis

## Abstract

Orthogonal experiments were designed for hybrid fiber rubber concrete (HFRC). The mechanical properties of HFRC were tested and compared with ordinary concrete. The effects of basalt fiber volume ratio (*V*_BF_), PVA fiber volume ratio (*V*_PF_) and rubber volume ratio (*V*_R_) on the compressive strength, splitting tensile strength and flexural strength of HFRC were analyzed. The results show that the strength of HFRC is the best when the volume ratio of basalt fiber is 0.3%, the volume ratio of PVA fiber is 0.2% and the volume ratio of rubber is 5%. Basalt fiber has the greatest influence on the strength of HFRC. The strength of HFRC mixed with hybrid fiber is greatly improved, which reflects the good fiber “positive hybrid effect”. With the increase of rubber volume ratio, the strength of HFRC decreases gradually. With the help of SEM and EDS, the toughening and cracking resistance mechanism of the fiber to HFRC was analyzed. Finally, the strength of HFRC was predicted by model.

## 1. Introduction

Concrete has the characteristics of high compressive strength and a wide range of strength grades. It has become the most widely used artificial building material in the world. At the same time, with the rapid development of global infrastructure, the application of concrete materials has become more and more extensive, which promotes the development of concrete in the directions of high strength, sustainable development, intelligence, etc. However, with the increasing requirements of construction engineering on all aspects of concrete, and the defects of ordinary concrete such as easy cracking, low tensile strength, poor toughness and heavy self-weight, it not only limits the exertion of the advantages of concrete to a great extent, but also reduces the service life of concrete structure. For a long time, from the perspective of improving the performance of concrete, many researchers have carried out a lot of research on improving the easy cracking, low tensile strength and poor toughness of concrete. Among them, mixing different admixtures into concrete has become one of the most effective ways [1,2,3,4].

With the rapid development of economic society, the global automobile production has increased rapidly, and automobile update frequency is also increasingly accelerated, leading to a sharp increase in the amount of discarded tires every year. Because discarded tires (solid polymers) are not easily degraded, they have caused a lot of “black pollution” to the environment. Therefore, the recycling and reuse of waste tires has become an urgent problem to be solved [5,6,7]. Crushing waste tires into rubber powder or rubber particles can replace part of the fine aggregate in concrete to provide rubber concrete [8]. On the one hand, rubber concrete has better fracture resistance, impact resistance, sound insulation and heat insulation performance than ordinary concrete [9]. The application of rubber concrete can alleviate the environmental pollution caused by discarded tires to a certain extent, and has broad application prospects [10]. Liu Yushan et al. [11] studied the fatigue resistance of rubber concrete in a dry-wet alternating environment and found that rubber concrete showed better elasticity than ordinary concrete. Zhang Juntao et al. [12] studied the frost resistance of rubber concrete under the action of freeze-thaw cycles. The results show that rubber concrete has better frost resistance, under the same load, the peak strength of rubber concrete decreases to a small extent, and the quality loss is not obvious. Hu Yanli et al. [13] conducted uniaxial compression and uniaxial splitting tests on rubber concrete with different rubber content. The results showed that rubber concrete can maintain good integrity under uniaxial compression failure, and rubber concrete has stronger plastic deformation capacity compared with ordinary concrete. However, as an organic material, rubber particles have a poor bonding force to the cement matrix. The increase in the content of rubber particles leads to an increase in internal defects of the matrix and a decrease in the mechanical properties of concrete. The research of Ganjian et al. [14] showed that the compressive and tensile strength of rubber concrete was decreased by replacing aggregates with rubber particles. The test results of Feng Lingyun et al. [15] showed that the rubber content was negatively correlated with the strength of concrete, but positively correlated with the change of its tension-compression ratio.

Studies have shown that fiber in concrete can better enhance the strength of concrete and make up for its brittle failure defects [16,17]. Basalt fiber is cheap, has a high elastic modulus and ultimate strain, and belongs to a new type of fiber with excellent performance, low cost and environmental protection. Although the elastic modulus and ultimate strain of PVA fiber are lower than basalt fiber, the elongation at break is greater than basalt fiber. The research [18,19,20,21] shows that the right amount of basalt fiber can improve the mechanical properties of concrete, obviously improve the toughness and failure mode of concrete, and improve the toughness index and fracture energy of concrete. PVA fiber can effectively improve the impermeability, corrosion resistance and frost resistance of concrete, and reduce the thermal expansion of concrete. On the other hand, the improvement in the concrete performance of one kind of fiber has great limitations, and the hybrid of two kinds of fiber can complement each other’s advantages and better provide the “positive hybrid effect” [22,23].

In this paper, a new type of hybrid fiber rubber concrete (HFRC) was prepared by mixing basalt-PVA hybrid fiber into rubber concrete to study the effects of the basalt-PVA hybrid fiber on the compressive strength, splitting tensile strength, flexural strength and impact resistance of HFRC, in order to provide reference and theoretical support for large-scale application of HFRC.

## 2. Raw Materials and Test Methods

### 2.1. Raw Materials

The cementitious material used in the test is Chinese standard Portland cement, and the 28d compressive strength is 42.5 MPa. The physical and mechanical properties of basalt fiber and PVA fiber are shown in Table 1. The particle size of rubber is 20 mesh and the density is 1050 kg/m^3^. The coarse aggregate used in the test is 5–25 mm continuously graded granite gravel, with a bulk density of 1460 kg/m^3^, an apparent density of 2660 kg/m^3^, a crushing index of 10.5% and a mud content of 0.5%. The fine aggregate used in the test is natural river sand with an apparent density of 2525 kg/m^3^ and a fineness modulus of 2.75. The water-reducing agent adopts HPWR liquid type high-performance water-reducing agent, and the water-reduction rate is 37%. The water is ordinary tap water.

### 2.2. Experimental Design

Japanese statistician Genichi Taguchi proposed an orthogonal table based on an orthogonal optimization scheme. When there are many test groups in the whole experiment, selecting representative points from the whole test according to the orthogonality can effectively reduce the number of tests, and the orthogonal experiment is dispersibility and neat comparability. The range and variance was applied to analyze the experimental data; by analyzing the degree of influence of variables on the research object, the best conditions or the best combination can be used to achieve the experimental goals [24,25,26]. In order to study the influence rule of basalt fiber volume rate-*V_BF_* (factor A), PVA fiber volume rate-*V_PF_* (factor B) and rubber volume rate-*V_R_* (factor C) on the compressive strength, splitting tensile strength and flexural strength of HFRC, the L9 (3^3^) orthogonal experiment scheme was selected, with each factor taking three levels. The factors and levels are shown in Table 2. According to JGJ 55-2011 ‘*Specification for mix proportion design of ordinary concrete*’, the concrete mix proportion design is shown in Table 3.

### 2.3. Sample Preparation

Because the fibers agglomerate easily in the process of specimen making, the main function of sodium carboxymethyl cellulose is to fully disperse the fiber in the concrete and make the fibers into a monofilament state. At the same time, this will not cause any impact on concrete. Therefore, 0.7% of the total mass of the cement is mixed with sodium carboxymethyl cellulose to improve the reinforcing effect of the fiber on the concrete in the sample preparation [27]. The test piece is made with a single-shaft horizontal concrete mixer. First, the coarse aggregate, fine aggregate, basalt fiber, PVA fiber, rubber and sodium carboxymethyl cellulose are mixed and dry mixed for 2 min, then the cement is poured in and dry mixed for 2 min, and finally water dissolved in water-reducer agent is added and stirred for 3 min to complete HFRC. For compressive strength and splitting tensile strength tests, three specimens of 100 mm × 100 mm × 100 mm cube specimens are made for each group, and three specimens of 100 mm × 100 mm × 400 mm strip specimens are made for each group of flexural strength test and cured in a non-flowing saturated calcium hydroxide solution at room temperature of 20 ± 2 °C for 28 days. The values of compressive strength, splitting tensile strength and flexural strength are the average values of the three test results of each group of specimens.

**Figure 1:**
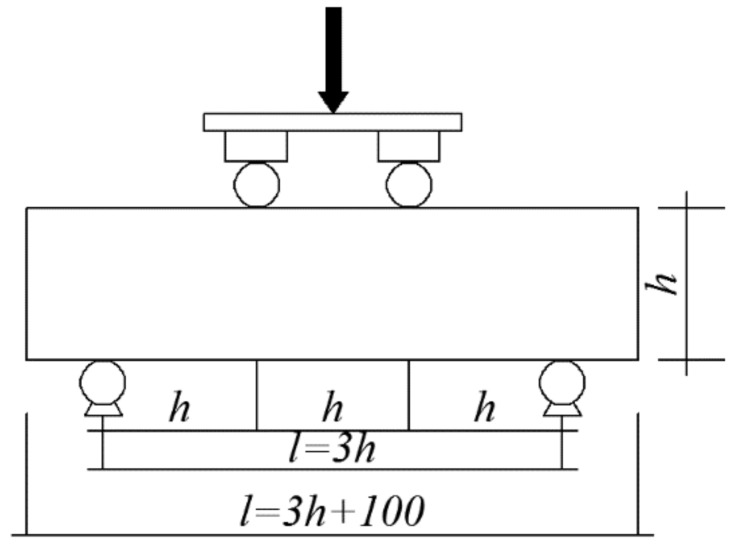
Loading device diagram of flexural strength test specimen.

According to the relevant provisions of the National Standard of the People’s Republic of China GB/T 50081-2019 *Standard for test methods for concrete physical and mechanical properties*, the compressive strength, splitting tensile strength and flexural strength are tested with a WAW-2000D electro-hydraulic servo universal testing machine. The calculation formula of the compressive strength value is shown in Equation (1), the splitting tensile strength value is shown in Equation (2), and the flexural strength is shown in Equation (3). The loading device diagram of the flexural strength test specimen is shown in Figure 1.

(1) $$f_{cc}=\frac{F}{A}$$

In Equation (1): *f_cc_* is the cubic compressive strength of the test piece (MPa); *F* is the failure load of the test piece (N); *A* is the pressure-bearing area of the test piece (mm^2^).

(2) $$f_{ts}=\frac{2F}{\pi A}=0.637\frac{F}{A}$$

In Equation (2): *f_ts_* is the splitting tensile strength of the test piece (MPa); *F* is the failure load of the test piece (N); *A* is the pressure-bearing area of the test piece (mm^2^).

(3) $$f_{f}=\frac{Fl}{bh^{2}}$$

In Equation (3): *f_f_* is the flexural strength of the test piece (MPa); *F* is the failure load of the test piece (N); *l* is the span between supports (mm); *b* is the section width of the test piece (mm); *h* is the section height of the test piece (mm).

Since the size of the test piece used to determine the compressive strength and splitting tensile strength is non-standard size specimens of 100 mm × 100 mm × 100 mm and the flexural strength test piece is non-standard size specimens of 100 mm × 100 mm × 400 mm. According to the relevant provisions of the National Standard of the People’s Republic of China GB/T 50081-2019 *Standard for test methods for concrete physical and mechanical properties*, the compressive strength, splitting tensile strength and flexural strength are converted into standard specimen results by multiplying the size conversion coefficient. The size conversion coefficient of the cube compressive strength is 0.95, and the size conversion coefficient of the splitting tensile strength and flexural strength is 0.85.

### 2.4. Data Processing

In order to study the effects of *V*_BF_ and *V*_PF_ on the compressive strength, splitting tensile strength and flexural strength of rubber concrete and find out the significant factors, the range and variance of the test results of compressive strength, splitting tensile strength and flexural strength of HFRC are calculated. The calculation method is shown in Equation (4):

Range calculation method:

(4) $$R_{i}=\max({\bar{K}}_{i1},{\bar{K}}_{i2},\ldots ,{\bar{K}}_{im})-\min({\bar{K}}_{i1},{\bar{K}}_{i2},\ldots ,{\bar{K}}_{im})(i=A,B,C;m=1,2,3,4)$$

In Equation (4): *K_im_* is the sum of the test results corresponding to the level of factor m in column *i*, ${\bar{K}}_{im}$ is the average value of *K_im_*, and *R_i_* is the range of factors in column *i*. The primary and secondary relationships of the factors are judged according to *R_i_*.

The variance is calculated as follows:

Sum of squares of total deviations:

(5) $$SST=\sum_{i=1}^{n}(y_{i}-\bar{y}{)}^{2}$$

Degree of freedom:

(6) $$f_{t}=n-1$$

In Equations (5) and (6): *n* is the number of rows (test times) of the orthogonal experiment table, and $\bar{y}$ is the average value of *n* test indexes.

Sum of square of deviations of factor A:

(7) $$SSA=\sum_{i=1}^{a}n_{i}{\left({\bar{y}}_{i}-\bar{y}\right)}^{2}$$

Degree of freedom:

(8) $$f_{A}=n_{i}-1$$

Mean square of factor A:

(9) $$MSA=\frac{SSA}{f_{A}}$$

In Equations (7)–(9): *A* is the level number of factor A, *n_i_* is the number of tests at level *i*, ${\bar{y}}_{i}$ is the average value of index at each level of factor A. Similar to the solution of the sum of the square sum of deviation, degree of freedom and mean square of factor A, the values of *SSB*, *SSC*, *f_B_*, *f_C_*, *MSB* and *MSC* can be obtained.

Sum of the squares of deviation of error:

(10) SSE=SST−SSA−SSB−SSC

Mean square of error:

(11) $$MSE=\frac{SSE}{f_{E}}$$

Significant level of factor A:

(12) $$F_{A}=\frac{MSA}{MSE}$$

Similar to the significance level of factor A, *F_B_* and *F_C_* can be obtained.

## 3. Experimental Results and Analysis

### 3.1. Experimental Result

The test results of the compressive strength, splitting tensile strength and flexural strength of each group of specimens are shown in Table 4. Based on the test results in Table 4, it can be seen intuitively that the compressive strength, splitting tensile strength and flexural strength of HFRC are greater than those of ordinary concrete, which indicates that the addition of basalt fiber, PVA fiber and rubber into concrete improves its mechanical properties. The intuitive analysis of the test results of 9 groups of HFRC shows that the mechanical properties of H-8 group are the best, that is, when *V*_BF_ is 0.3%, *V*_PF_ is 0.2%, and *V*_R_ is 5%, the compressive strength, splitting tensile strength and flexural strength of HFRC reached the peak value of 47.9 MPa, 6.63 MPa and 8.98 MPa, respectively. Compared with ordinary concrete, the three kinds of strength increase of H-8 group are 40.06%, 42.58% and 37.3%, respectively. This shows that *V*_BF_,*V*_PF_ and *V*_R_ have the greatest impact on the splitting tensile strength of HFRC.

### 3.2. Range Analysis

**Figure 2:**
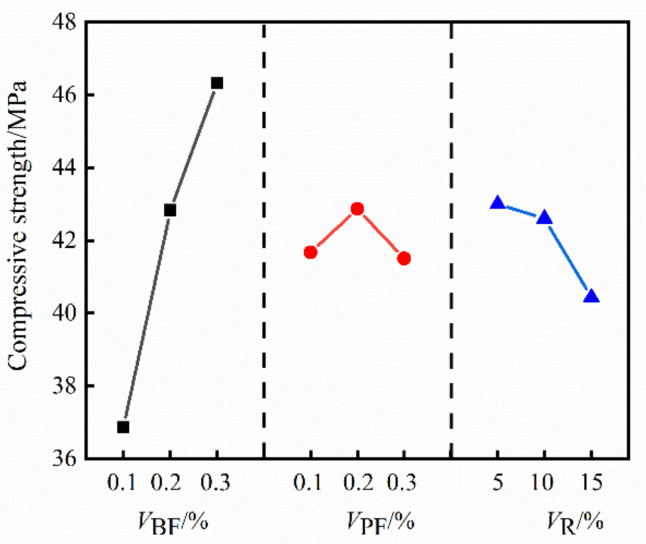
The impact trend of *V*_BF_, *V*_PF_ and *V*_R_ on the compressive strength of HFRC.

**Figure 3:**
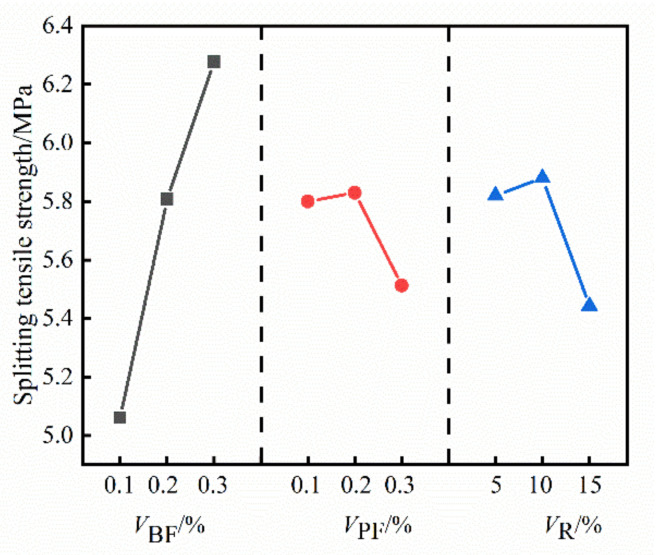
The impact trend of *V*_BF_, *V*_PF_ and *V*_R_ on the splitting tensile strength of HFRC.

**Figure 4:**
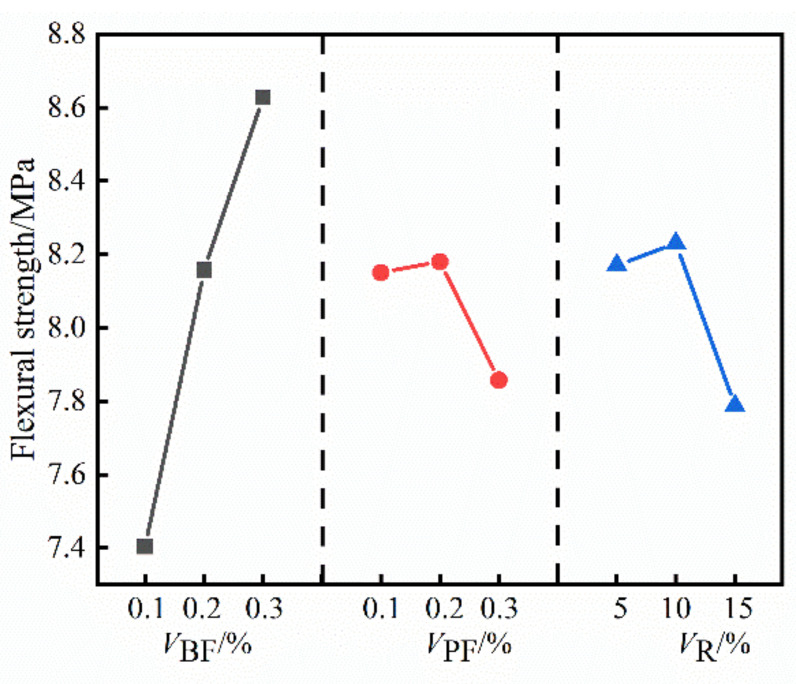
The impact trend of *V*_BF_, *V*_PF_ and *V*_R_ on the flexural strength of HFRC.

The range of the compressive strength, splitting tensile strength and flexural strength of HFRC in Table 4 was calculated, and the results are shown in Table 5. The impact trend of *V*_BF_,*V*_PF_ and *V*_R_ on the compressive strength of HFRC is shown in Figure 2, the impact trend of *V*_BF_,*V*_PF_ and *V*_R_ on the splitting tensile strength of HFRC is shown in Figure 3, and the impact trend of *V*_BF_,*V*_PF_ and *V*_R_ on the flexural strength of HFRC is shown in Figure 4.

It can be seen from Table 5 that the effect size of *V*_BF_,*V*_PF_ and *V*_R_ on the compressive strength, splitting tensile strength and flexural strength of HFRC are all *V*_BF_ > *V*_R_ > *V*_PF_.

It can be seen from Figure 2, Figure 3 and Figure 4 that the compressive strength, splitting tensile strength and flexural strength of HFRC gradually increase with the increase of *V*_BF_. When *V*_BF_ increased from 0.1% to 0.3%, the compressive strength, splitting tensile strength and flexural strength of HFRC increased by 25.66%, 24.05% and 16.53%, respectively. With the increase of *V*_PF_, the compressive strength, splitting tensile strength and flexural strength of HFRC increased first and then decreased; when *V*_PF_ increased from 0.1% to 0.2%, the compressive strength, splitting tensile strength and flexural strength of HFRC increased by 2.88%, 0.52% and 0.37%, respectively; when *V*_PF_ increased from 0.1% to 0.3%, the three strengths of HFRC decreased by 0.41%, 4.95% and 3.6%, respectively. With the increase of *V*_R_, the compressive strength of HFRC gradually decreased, the splitting tensile strength and flexural strength showed a trend of first increasing and then decreasing; when *V*_R_ increased from 5% to 10%, the splitting tensile strength and flexural strength of HFRC increased by 1.03% and 0.61%, respectively; when *V*_R_ increased from 5% to 15%, the compressive strength, splitting tensile strength and flexural strength of HFRC decreased by 5.98%, 6.48% and 4.69%, respectively.

### 3.3. Variance Analysis

The variances of compressive strength, splitting tensile strength and flexural strength of HFRC in Table 4 are calculated, and the results are shown in Table 6.

Based on the variance analysis of the compressive strength, splitting tensile strength and flexural strength of HFRC in Table 6, it can be seen that *V*_BF_ is an extremely significant factor affecting the three strengths of HFRC; *V*_PF_ has a certain impact on the compressive strength of HFRC and *V*_PF_ represents non-significant factors affecting the splitting tensile strength and flexural strength of HFRC; *V*_R_ is a significant factor affecting the compressive strength of HFRC, and the impact on the splitting tensile strength and flexural strength of HFRC is relatively close. All showed a certain impact, but the impact on the flexural strength is greater than the splitting tensile strength.

### 3.4. Analysis of Efficacy Coefficient

The above is the range and variance analysis of the compressive strength, splitting tensile strength and flexural strength of HFRC, but it is not a comprehensive evaluation of the mechanical properties of HFRC. The compressive strength, splitting tensile strength and flexural strength of HFRC in Table 4 were comprehensively evaluated by the efficacy coefficient method. The maximum and minimum values of the three strengths of HFRC were selected as the satisfactory and unsatisfactory values of the index evaluation system of the efficacy coefficient method, and the specific values are shown in Table 7.

The efficacy coefficient values of each index of 9 groups of HFRC were calculated according to the satisfactory and unsatisfactory values of each index, and the calculation formula is shown in Formula (13).

(13) $$d_{i}=\frac{\mathrm{Actual}\;\mathrm{value}-\mathrm{Unsatisfactory}\;\mathrm{value}}{\mathrm{Satisfactory}\;\mathrm{value}-\mathrm{Unallowable}\;\mathrm{value}}\times 40+60(i=1,2,3)$$

In Formula (13): *d*_1_ represents the efficiency coefficient value of each group of the compressive strength of HFRC, *d*_2_ represents the efficiency coefficient value of each group of the splitting tensile strength of HFRC, and *d*_3_ represents the efficiency coefficient value of each group of the flexural strength of HFRC.

Total efficacy coefficient value of each group of HFRC:

(14) $$d=\sqrt[{3}]{d_{1}\times d_{2}\times d_{3}}$$

**Figure 5:**
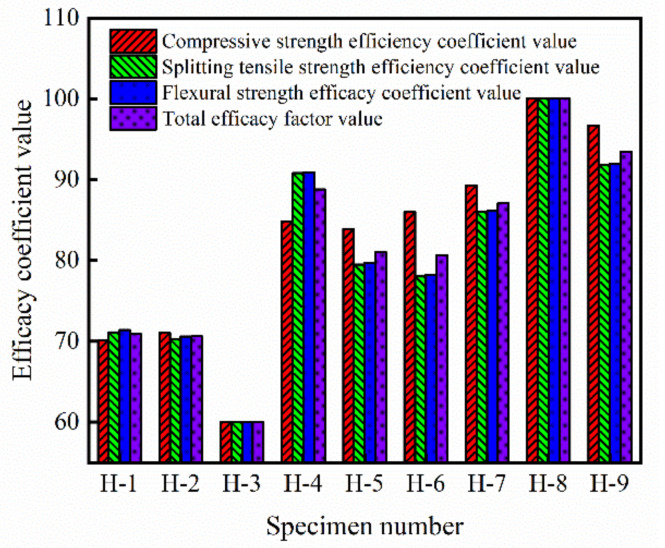
The efficiency coefficient value of HFRC.

Figure 5 shows the calculation results of the compressive strength efficiency coefficient value, splitting tensile strength efficiency coefficient value, flexural strength efficiency coefficient value and total efficiency coefficient value of each group of HFRC specimens. It can be seen that the total efficacy coefficient value of group H-8 specimens is the largest, which is 100. Therefore, of the three indexes of compressive strength, splitting tensile strength and flexural strength, the best mix proportion combination is A_3_B_2_C_1_, that is, *V*_BF_ is 0.3%, *V*_PF_ is 0.2% and *V*_R_ is 5%.

## 4. Analysis of SEM Micro

The stress of the concrete in the initial stage of bearing the external load is mainly borne by the HFRC matrix. At this time, the stress is small, but with the continuous increase of load, tiny cracks begin to appear in the concrete and slowly develop into cracks that penetrate the whole specimen. At this time, basaltic fiber with high elastic modulus bears the main load through mechanical bite force and bonding force with concrete matrix and plays the reinforcement role of basalt fiber. With the continuous increase of load, when small cracks develop into larger cracks, the adhesive force formed by the bridging action between the basalt fiber and the concrete matrix will become much smaller. At this time, the PVA fiber begins to interact with the basalt fiber after compression deformation, which enhances the strength of the concrete matrix synergistically, and reflects the “positive hybrid effect” of the fiber. However, due to the low elastic modulus of PVA fiber, the reinforcement effect on concrete is limited, so the reinforcement effect of PVA fiber is much smaller than that of basalt fiber. Mixing rubber into concrete can improve the workability of HFRC. From the previous range analysis and variance analysis of the three strengths of HFRC, it can be seen that: (1) Rubber is not a significant factor affecting the strength of HFRC. (2) Mixing a certain amount of rubber into concrete can improve the strength of concrete to a certain extent, but the effect is not obvious and the strength of concrete even shows a negative increase after exceeding the amount.

**Figure 6:**
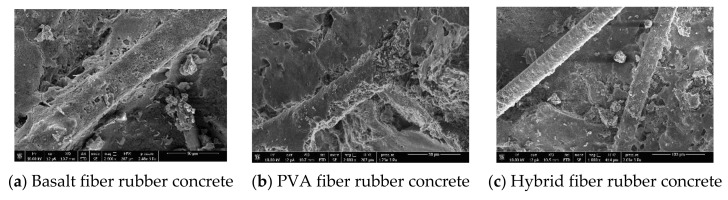
Adhesion between fiber and matrix.

As can be seen from Figure 6, dense spherical material and thorn-like cement hydration matrix (C-S-H gel) cover the outer surface of the fiber. When HFRC undergoes the cement hydration reaction, ettringite, a secondary hydration product, will be formed. Once HFRC bears an external load, C-S-H gel and ettringite are tightly wrapped around the fiber, and the fiber can form a certain bond with the HFRC matrix, which plays a role in strengthening the HFRC. The fibers are distributed in the form of a three-dimensional grid in the HFRC, which reduces the stress concentration at the HFRC cracks and plays a role in preventing the generation and expansion of the cracks.

## 5. EDS Test

**Figure 7:**
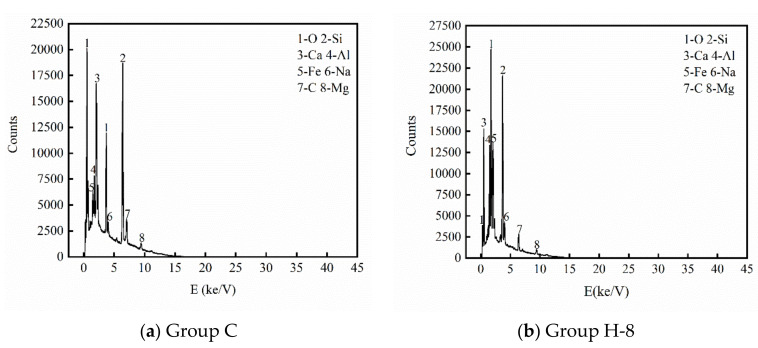
EDS text of HFRC.

In order to intensively study the mechanism of action of basalt-PVA fiber reinforced HPBC, group C and group H-8 were selected for EDS test and quantitative analysis. Figure 7 shows the EDS test results of the C group and H-8 group, and Table 8 shows the EDS quantitative analysis results of the two groups of concrete. It can be seen that the element types of the C group and the H-8 group are the same. The obvious difference is that the EDS peak value of O, Si, and Ca elements are quite different, and the peak value of group H-8 is more prominent than that of group C. This shows that due to the synergistic effect of basalt-PVA fiber, the cement hydration reaction of group H-8 is better and effectively enhances the strength of rubber concrete.

## 6. Strength Prediction Model

According to the theory of material mechanics, it is assumed that the strength of HFRC is composed of concrete matrix strength, basalt fiber reinforcement, PVA fiber reinforcement and rubber reinforcement. The strength regression model is assumed to be:

(15) $$f=\beta_{0}+\beta_{1}x_{1}+\beta_{2}x_{2}+\beta_{3}x_{3}+\alpha$$

In Equation (15): *f* is the compressive strength, splitting tensile strength or flexural strength of concrete (MPa); *β*_0_ is the compressive strength, splitting tensile strength or flexural strength of concrete matrix (MPa); *β*_1_, *β*_2_, *β*_3_ is the regression coefficient; α is the test parameter; *x*_1_ = *V*_BF_ (%); *x*_2_ = *V*_PF_ (%); *x*_3_ = *V*_R_ (%).

The data in Table 4 are substituted into the regression model (15), and the regression equation of compressive strength splitting tensile strength and flexural strength of HFRC is estimated by the least square method [28] for *β*.

(16) $$f_{cc}=36.28+4.733x_{1}-0.083x_{2}-1.283x_{3}(R^{2}=0.94)$$

(17) $$f_{ts}=5.161+0.608x_{1}-0.143x_{2}-0.188x_{3}(R^{2}=0.89)$$

(18) $$f_{f}=7.516+0.612x_{1}-0.147x_{2}-0.192x_{3}(R^{2}=0.89)$$

In Equations (16)–(18): *f_cc_* is the compressive strength of HFRC (MPa); *f_ts_* is the splitting tensile strength of HFRC (MPa); *f_f_* is the flexural strength of HFRC (MPa); *R^2^* is the determining coefficient.

**Figure 8:**
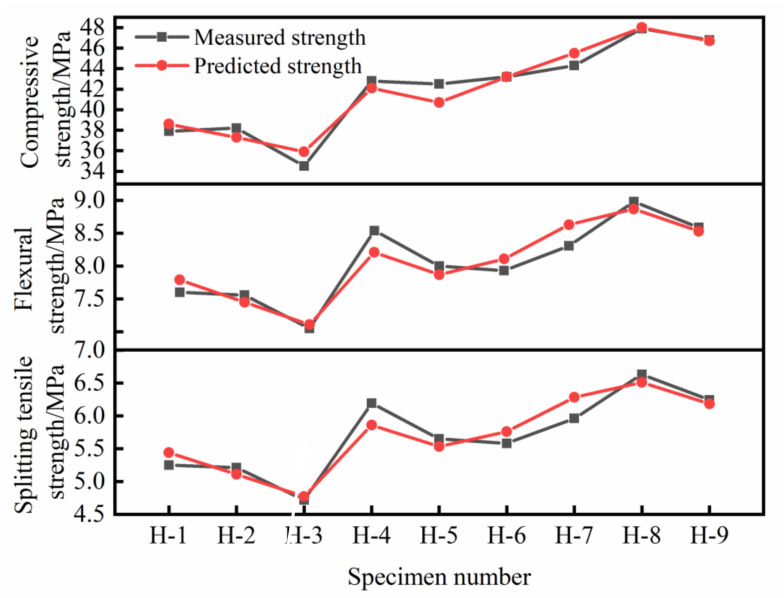
The predicted value and the measured value of HFRC strength.

Figure 8 is the comparison between the predicted value and the measured value of HFRC strength. It can be seen that the maximum errors are all within 12%, indicating that the model has good accuracy and can provide a certain reference value for engineering construction.

## 7. Conclusions

1. The compressive strength, splitting tensile strength and flexural strength of HFRC are greater than those of ordinary concrete; the compressive strength is between 34.5–47.9 MP, the splitting tensile strength is between 4.72–6.63 MPa, and the flexural strength is between 7.05–8.98 MPa, indicating that basalt fiber and PVA fiber improve the strength of rubber concrete.
2. The influence degree of the three factors on the compressive strength, splitting tensile strength and flexural strength of HFRC is *V*_BF_ > *V*_R_ > *V*_PF_. When *V*_BF_ increased from 0.1% to 0.3%, the compressive strength, splitting tensile strength and flexural strength of HFRC increased by 25.85%, 23.59% and 16.31%, respectively.
3. *V*_BF_ is an extremely significant factor affecting the strength of HFRC; *V*_PF_ has a certain impact on the compressive strength of HFRC and is an non-significant factor of the splitting tensile strength and flexural strength of HFRC; *V*_R_ is a significant factor affecting the compressive strength of HFRC and it also has a certain impact on the splitting tensile strength and flexural strength of HFRC, but the impact on the flexural strength is greater than that on the splitting tensile strength.
4. The hybrid of basalt fiber and PVA fiber showed a good synergy effect of fiber to enhance the strength of HFRC and reflected a good “positive hybrid effect” of fiber. In the studied HFRC in this paper, when the volume ratio of rubber is 5%, the increase in its strength has a certain effect, but when the volume ratio of rubber exceeds 5%, the strength of HFRC will show a negative growth effect.
5. The best mix proportion combination is A_3_B_2_C_1_, that is, *V*_BF_ is 0.3%, *V*_PF_ is 0.2%, and *V*_R_ is 5%. The bridging and reinforcement effect of fiber in HFRC was analyzed with the help of SEM microscopic and quantitative analysis of EDS. Finally, the strength of HFRC was predicted.

## Figures and Tables

**Table 1:** Physical and mechanical properties of basalt fiber and PVA fiber.

Fiber	Basalt Fiber	PVA Fiber
Tensile strength/MPa	3500	600
Modulus of elasticity/GPa	100	5.75
Density/(g/cm^3^)	2.8	0.91
Diameter/µm	12	8
Length/mm	18	12
Elongation at break/%	3.2	16

**Table 2:** Factors and levels table.

Level	Factor		
	*V_BF_*/(%)	*V_PF_*/(%)	*V_R_*/(%)
1	0.1	0.1	5
2	0.2	0.2	10
3	0.3	0.3	15

**Table 3:** Mix proportion of HFRC kg/m^3.^

Specimen Number	Cement	Coarse Aggregate	Fine Aggregate	Basalt Fiber	PVA Fiber	Rubber	Water	Water-Reducing Agent
C	465	1242	600	0	0	0	155	5.15
H-1	465	1242	600	2.8	0.91	52.5	155	5.15
H-2	465	1242	600	2.8	1.82	105	155	5.15
H-3	465	1242	600	2.8	2.73	157.5	155	5.15
H-4	465	1242	600	5.6	0.91	105	155	5.15
H-5	465	1242	600	5.6	1.82	157.5	155	5.15
H-6	465	1242	600	5.6	2.73	52.5	155	5.15
H-7	465	1242	600	8.4	0.91	157.5	155	5.15
H-8	465	1242	600	8.4	1.82	52.5	155	5.15
H-9	465	1242	600	8.4	2.73	105	155	5.15

**Table 4:** Test results of compressive strength, splitting tensile strength and flexural strength of each group of specimens.

Specimen Number	Compressive Strength /MPa	Splitting Tensile Strength /MPa	Flexural Strength /MPa
C	34.2	4.65	6.54
H-1	37.9	5.25	7.6
H-2	38.2	5.21	7.56
H-3	34.5	4.72	7.05
H-4	42.8	6.19	8.54
H-5	42.5	5.65	8.0
H-6	43.2	5.58	7.93
H-7	44.3	5.96	8.31
H-8	47.9	6.63	8.98
H-9	46.8	6.24	8.59

**Table 5:** Calculation results of the range of HFRC strength.

Factor	Compressive Strength			Splitting Tensile Strength			Flexural Strength		
	*V*_BF_	*V*_PF_	*V*_R_	*V*_BF_	*V*_PF_	*V*_R_	*V*_BF_	*V*_PF_	*V*_R_
R	9.47	1.37	2.57	1.217	0.317	0.437	1.223	0.323	0.443

**Table 6:** Calculation result of variance of HFRC strength.

Index	Factor	Sum of Squares	Mean Square	Freedom	F value	Significance
Compressive strength/MPa	A	137.47	68.73	2	190.34	**
	B	3.34	1.67	2	4.62	⦿
	C	11.44	5.72	2	15.84	*
	E	0.72	0.36	2		
Splitting tensile strength/MP	A	2.26	1.13	2	27.71	**
	B	0.18	0.09	2	1.76	_
	C	0.34	0.17	2	3.23	⦿
	E	0.1	0.05	2		
Flexural strength/MPa	A	2.28	1.14	2	23.12	**
	B	0.19	0.1	2	1.94	_
	C	0.35	0.17	2	3.51	⦿
	E	0.1	0.05	2		

Notes: ** represents extremely significant, * represents significant, ⦿ represents a certain impact, and _ represents non-significant.

**Table 7:** Satisfaction and unsatisfactory values of each index.

Value Type	Compressive Strength /MPa	Splitting Tensile Strength/ MPa	Flexural Strength /MPa
Satisfactory value	47.9	6.63	8.98
Unsatisfactory value	34.5	4.72	7.05

**Table 8:** EDS quantitative analysis of HFRC.

Element	Group C		Group H-8	
	Mass Content /%	Atomic Content /%	Mass Content /%	Atomic Content /%
O	33.67	50.49	46.68	62.65
Si	12.71	10.86	23.25	17.77
Ca	26.7	15.98	19.94	10.68
Al	6.32	5.61	4.39	3.49
Fe	7.79	3.34	1.75	0.67
Na	0.72	0.75	1.01	0.95
C	3.11	6.21	1.55	2.77
Mg	0.85	0.84	0.72	0.64

## Data Availability

The data used to support the findings of this study are included within the article.
